# Novel High-Throughput Multiplex qPCRs for the Detection of Canine Vector-Borne Pathogens in the Asia-Pacific

**DOI:** 10.3390/microorganisms9051092

**Published:** 2021-05-19

**Authors:** Lucas Huggins, Luca Massetti, Bettina Schunack, Vito Colella, Rebecca Traub

**Affiliations:** 1 Faculty of Veterinary and Agricultural Sciences, University of Melbourne, Parkville, VIC 3052, Australia; lmassetti@student.unimelb.edu.au (L.M.); vito.colella@unimelb.edu.au (V.C.); rebecca.traub@unimelb.edu.au (R.T.); 2 Elanco GmbH, Heinz-Lohmann-Str. 4, 27472 Cuxhaven, Germany; bettina.schunack@elancoah.com

**Keywords:** multiplex qPCR, canine vector-borne disease, dogs, molecular diagnostics, Asia-Pacific, *Anaplasma* *platys*, *Babesia*, *Ehrlichia* *canis*, *Hepatozoon* *canis*, haemotropic *Mycoplasma*

## Abstract

The Asia-Pacific hosts a large diversity of canine vector-borne pathogens (VBPs) with some of the most common and most pathogenic, generating significant mortality as well as a spectrum of health impacts on local dog populations. The VBPs *Anaplasma platys*, *Babesia gibsoni*, *Babesia vogeli*, *Ehrlichia canis*, *Hepatozoon canis* and haemotropic *Mycoplasma* spp. are all endemic throughout the region, with many exhibiting shifting geographical distributions that warrant urgent attention. Moreover, many of these species cause similar clinical signs when parasitising canine hosts, whilst knowledge of the exact pathogen is critical to ensure treatment is effective. This is complicated by frequent coinfection that can exacerbate pathology. Here, we describe the development, optimisation and validation of two novel quadruplex Taq-Man based real-time PCRs (qPCRs) for the specific and sensitive detection of the aforementioned VBPs. To ensure accurate evaluation of diagnostic performance, results of our qPCRs were evaluated on field samples from Thai dogs and compared with both conventional PCR (cPCR) results and next-generation sequencing (NGS) metabarcoding. Our qPCRs were found to be more sensitive at detecting canine VBP than cPCR and generated results similar to those achieved by NGS. These qPCRs will provide a valuable high-throughput diagnostic tool available to epidemiologists, researchers and clinicians for the diagnosis of key canine VBPs in the Asia-Pacific and further afield.

## 1. Introduction

A plethora of pathogenic agents ranging in size from minute arboviruses to bacteria, protozoa, and multicellular parasites, such as filarial nematodes, can all be transmitted by blood-feeding arthropods and are collectively known as vector-borne pathogens (VBPs) [1,2]. VBPs due to their vectors’ ubiquitous nature are found globally and affect a vast range of animals including humans. This has been highlighted through outbreaks of diseases like Zika [3], Dengue Fever [4], leishmaniasis [5], Chagas disease [6] and tick-borne diseases, for example Lyme disease [7]. Dogs are a host species that are known to acquire an expansive variety of VBPs, producing a spectrum of disease from acute and potentially fatal infections, to chronic and recrudescing illness that may be carried by the canine host for the duration of its life [8]. Pathogenesis typically centres around non-specific clinical signs, including fever, anaemia, thrombocytopenia, anorexia and lethargy that make precise diagnosis of the aetiological agent essential for administration of effective treatment [8,9,10].

In the tropics canine VBP prevalence can be especially high, due to climactic conditions that encourage the fast growth and reproduction of the one-host brown dog tick, *Rhipicephalus sanguineus* sensu lato, an important vector of numerous pathogens [11]. One key phylum from which many canine VBPs belong to is the Apicomplexa, which includes the species *Babesia vogeli*, *Hepatozoon canis* and less commonly in the tropics, *Babesia gibsoni* [11,12,13]. *Babesia* parasites infect erythrocytes after arthropod blood-feeding, whilst *H. canis* is transmitted by host ingestion of an infected tick [14,15,16]. Apicomplexan VBPs produce a range of disease severity in parasitised dogs, from the potentially fatal *B. gibsoni* to the less virulent *B. vogeli* that is more pathogenic in younger canines. In contrast, hepatozoonosis is often asymptomatic, although in a small percentage of individuals unable to control the infection, high levels of parasitaemia can develop, accompanied by cachexia, anaemia and even death [16,17,18].

Bacterial vector-borne disease, such as those produced by the rickettsial organisms *Anaplasma platys* and *Ehrlichia canis* are also highly abundant in dogs in the tropics causing canine cyclic thrombocytopenia (CCT) and canine monocytic ehrlichiosis (CME), respectively [10,11,19,20]. CME can often be lethal in canines if left untreated, generating fever, anorexia, myalgia, bleeding tendencies, epistaxis and neurological complications [10,21]. Fatal outbreaks of *E. canis* have recently been discovered in an expanding range of arid and tropical regions of central and northern Australia, a country that had previously benefited from an *Ehrlichia*-free status until 2020 [21,22]. Haemotropic *Mycoplasma* species are also important VBPs of dogs and although their exact mechanisms of spread are still debated, tick-transmission is suspected to play a role, with these pathogens typically generating disease in splenectomised or immunocompromised dogs [23,24,25,26]. Moreover, many of these VBPs are commonly found coinfecting the same host, which can result in exacerbated disease and worse prognosis [27,28,29,30].

Given the breadth of potential canine VBPs, accurate diagnosis of the responsible species is critically important as each may require different treatment protocols to cure the infection [8,31]. Diagnosis has historically been conducted by microscopic examination of stained blood smears, a method notorious for its poor sensitivity, that also requires substantial expertise on the part of the microscopist to distinguish morphologically similar pathogens [9,32,33]. Some of these issues are also encountered when conducting serological diagnosis, e.g., low sensitivity or poor specificity due to antibody cross-reactivity [30]. Today, molecular techniques such as conventional and real-time PCR (qPCR) are regularly used for canine VBP diagnoses given their ability to detect low levels of circulating pathogen DNA, whilst at the same time accurately discerning different species [34,35]. Taq-Man based assays have been proven to be superior to conventional PCR and SYBR-based assays, especially in detecting mixed species infections, whilst also representing an ideal high-throughput diagnostic method suitable for large epidemiological studies [34,36,37,38].

In this study, we developed two multiplex Taq-Man qPCR assays to simultaneously detect the common canine VBPs circulating in the Asia-Pacific, i.e., *A. platys*, *B. gibsoni*, *B. vogeli*, *E. canis*, *H. canis* and haemotropic *Mycoplasma* spp. [11,12,18,39,40,41]. Herein, we define the Asia-Pacific as East Asia, including China, South Asia, including India, Southeast Asia and Oceania.

## 2. Materials and Methods

### 2.1. Multiplex qPCR Development

Our multiplex qPCR was designed to consist of two quadruplex reactions, i.e., one targeting the bacterial canine vector-borne pathogens *A. platys*, *E. canis*, haemotropic *Mycoplasma* spp. and a mammal mitochondrial DNA (mtDNA) as a DNA extraction control, whilst the other designed to target the apicomplexans *B. gibsoni*, *B. vogeli* and *H. canis* and DNA of Equine Herpes Virus (EHV) as an internal qPCR reaction control.

For the apicomplexan quadruplex designs *18S* ribosomal RNA (*18S rRNA*) gene sequences were downloaded from NCBI and aligned in Geneious Prime v. 2021.0.3. (Biomatters Ltd., Auckland, New Zealand), alongside sequences from other non-target canine apicomplexan pathogens to ensure specificity. All reference sequences of target and non-target organisms are shown in Supplementary File S1. A range of parasite isolate sequences found in different geographic regions were used to design probes against, whilst common non-target pathogens and environmental contaminant species’ sequences were also included. Given that some regions of the *18S* gene are highly conserved across the phylum Apicomplexa, regions that were identical across the three target species were investigated as potential primer binding sites, particularly if they flanked regions of diversity that could be used as probe binding sites. Putative regions were manually assessed, and then exact primer binding sites were determined using the open-source software Primer3 v. 0.4.0. (https://bioinfo.ut.ee/primer3-0.4.0/, accessed on 13 July 2020). Following this, primer and probe designs were further analysed and modified to ensure suitability in a multiplex format using the OligoAnalyzer™ Tool (Integrated DNA Technologies, Coralville, IA, USA), which permitted assessment of optimal annealing temperatures as well as removal of potential homo- and hetero-dimers from the designs. Finally, primers and probes were tested for specificity in silico via running NCBI’s BLASTn tool to confirm that high nucleotide identity was only achieved with the relevant target species. The same design process was conducted for the bacterial pathogen quadruplex assay, with the *16S rRNA* gene targeted instead. Reference sequences of target and non-target organisms are shown in Supplementary File S1 with final designs for both quadruplex assays in Supplementary File S2.

All primer and probe were designed using locked nucleic acid (LNA) technology to increase thermal stability and hybridization specificity. All qPCRs were run on a four-channel Magnetic Induction Cycler (Bio Molecular Systems, Upper Coomera, QLD, Australia).

For the apicomplexan quadruplex assay one primer pair Api-F and Api-R was used to amplify a common 180 bp stretch of the *18S rRNA* gene from *B. gibsoni*, *B. vogeli* and *H. canis*, with three separate Taq-Man probes (Table 1) as well as a previously designed EHV-4 primer pair and probe internal control amplifying an 80 bp region of the EHV-4 glycoprotein B (*gB*) gene [42]. For the bacterial quadruplex the primer pair Ehr/Ana-F and Ehr/Ana-R were designed to amplify a 145–149 bp region from both *A. platys* and *E. canis*, alongside two unique probes, whilst the primer pair Myco-F-D1 and Myco-R-Mod1 was designed to amplify a conserved 134 bp region of various haemotropic *Mycoplasma* spp. *16S rRNA* genes. A separate primer pair and probe taken and modified from [35], that target a 92 bp region of the canine mitochondrial *16S rRNA* gene were included in the bacterial quadruplex to act as a DNA extraction control (Table 1). Upon in silico examination of this primer pair and probe the mtDNA *16S rRNA* gene region targeted is conserved across a range of vertebrate species, including bears, monkeys, whales, otters, seals and snakes. Both quadruplex assays are designed to be run as a single panel; one includes the EHV internal reaction control, capable of detecting the presence of qPCR inhibitors, whilst the mtDNA extraction control in the bacterial quadruplex assesses whether the DNA extraction procedure has been successful and therefore if template DNA is present.

Optimisation to find ideal primer and probe concentrations was conducted using gBlock synthetic double-stranded DNA fragments (Integrated DNA Technologies) of the target region for each singleplex reaction. Serial dilution series of gBlock fragments used alongside iterative and stepwise testing of primer and probe concentrations permitted elucidation of the optimal concentration of each reagent (Table 1) to give the highest assay efficiency and greatest sensitivity.

We used QuantiNova Probe PCR Master Mix (Qiagen, Hilden, Germany) in the recommended 10 µL reactions for all quadruplex assays. Overall, for each quadruplex 10 μL reaction we used 1 μL of template with the addition of 1 μL of 1 × 10^−7^ ng/μL EHV-4 gDNA in the apicomplexan quadruplex, plus the relevant quantities of qPCR probes and primers listed in Table 1. Ambion Nuclease-Free Water (Life Technologies, Carlsbad, CA, USA) was used to achieve the final 10 μL reaction volume.

All reactions were conducted using the following thermocycling profile: 2 min at 95 °C, then 15 s at 95 °C and 1 min at 60 °C for 40 cycles, for both quadruplex assays. Synthetic gBlock fragments diluted to 1 × 10^−7^ ng/μL were included as positive controls for all pathogens tested as well as nuclease-free water as negative controls. Samples and controls were run in duplicate for all optimisation and testing. Cycling analysis parameters were set at a fluorescent threshold of 5% and the first five cycles for all pathogen targets were ignored. Twelve-fold serial dilutions of gBlock fragments for each pathogen with a known DNA concentration were made and used to assess the efficiency and the intra-assay reproducibility of singleplex reactions vs. when they were layered in a single multiplex reaction. The correlation coefficients (R^2^) from standard curves were produced and assessed using MIC PCR software (BioMolecular Systems, Upper Coomera, QLD, Australia).

### 2.2. Sensitivity and Specificity of Quadruplex qPCRs

Analytical sensitivity as defined by Saah and Hoover (1997) of the optimised multiplex assays was evaluated using 12-fold serial dilutions of gBlock synthetic DNA for each pathogen targeted [43]. Starting concentrations were 11.9 ng/μL for *B. gibsoni*, 10.8 ng/μL for *B. vogeli*, 7.5 ng/μL for *H. canis*, 2.86 ng/μL for *A. platys*, 7.78 ng/μL for *E. canis*, 5.24 ng/μL for *M. haemocanis* and 5.2 ng/μL for mammalian mtDNA with serial dilutions being made down to concentrations of 1 × 10^−12^ ng/μL.

The ability for our assay to exclusively detect our target pathogens and not detect similar but different organisms’ DNA, i.e., its analytical specificity [43], was achieved by testing the multiplex qPCRs against a bank of related and relevant blood-borne pathogens of zoonotic and veterinary importance. Potential cross-reactivity was tested using genomic DNA from animal blood or culture for the species *Anaplasma phagocytophilum*, *Bartonella clarridgeiae*, *Bartonella henselae*, *Borrelia burgdorferi*, *Coxiella burnetii*, *Orientia* spp., *Rickettsia australis*, *Rickettsia felis*, *Rickettsia typhi*, *Rickettsia honei*, *Leishmania infantum*, *Trypanosoma evansi* and *Dirofilaria immitis*. Both quadruplex assays were able to detect and amplify genomic DNA from their respective target organisms, with the haemotropic *Mycoplasma* probe specifically confirmed to detect genomic DNA from *M. haemocanis*, *Candidatus* Mycoplasma haematoparvum and *Candidatus* Mycoplasma turicensis. The possibility of cross-reactivity against pathogens targeted by the opposing quadruplex was also assessed, i.e., that the apicomplexans detected by the apicomplexan quadruplex were not detected by the bacterial quadruplex assay and vice versa.

The ability of our multiplex qPCRs to correctly classify an individual as being positive or negative for a particular pathogen, i.e., their diagnostic sensitivity and specificity [43], was ascertained using 100 samples previously characterised for canine VBP using both next-generation sequencing (NGS) metabarcoding and conventional PCR (cPCR). The results of these samples previously reported by Huggins et al. (2019a and 2019b) [13,20] were compared to those ascertained by the newly developed quadruplex assays.

For diagnostic sensitivity calculations the merged results of cPCR and NGS metabarcoding analysis were taken as a gold standard, i.e., samples positive by either cPCR and NGS for any given pathogen were considered true positives, whilst samples negative by both cPCR and NGS for any given pathogen were considered true negatives. Diagnostic sensitivity was calculated as the number of qPCR positives divided by the number of true positives (NGS and/or cPCR), whilst diagnostic specificity was taken as the number of qPCR negatives divided by the number of true negatives (NGS and cPCR).

### 2.3. Samples & DNA Extraction

Canine blood samples were collected from geographic locations known to be highly endemic for the canine VBPs targeted by our assay. One hundred samples collected as part of an ongoing research project with Kasetsart University, Thailand, and previously tested for a range of bacterial and apicomplexan pathogens using NGS metabarcoding and conventional PCR (cPCR) were used as a test cohort with which to compare diagnostic parameters of our multiplex qPCRs [13,20]. These samples were collected from stray dogs inhabiting pagoda communities across 35 different field sites, with a qualified veterinarian conducting blood sample collection through a cephalic or jugular puncture. A total of 1ml of blood was collected into EDTA tubes and stored at −20 °C until DNA extraction could be completed according to manufacturer’s instructions using a E.Z.N.A.^®^ Blood DNA Mini Kit (Omega Biotek Inc., Norcross, GA, USA), with DNA eluted in a total of 100 µL elution buffer. A further twelve samples were collected from stray dogs in Nanning, China, previously tested and confirmed positive for the pathogen *B. gibsoni*. Sample collection was only done after informed consent was acquired from dog owners, and the study was carried out under ethics permit OACKU-00758 provided by Kasetsart University.

### 2.4. Methodological Comparison and Statistical Analysis

Kappa statistics (*k*) used to compare agreement between the NGS and cPCR results vs. the qPCR multiplex assays, calculated in IBM SPSS Statistics 27 (SPSS, Chicago, IL, USA). Agreement between tests was considered poor if *k* ≤ 0.20, fair if 0.21 ≤ *k* ≤ 0.40, moderate if 0.41 ≤ *k* ≤ 0.60, substantial if 0.61 ≤ *k* ≤ 0.80 and high if *k* > 0.81. Endpoint PCRs were used as another reference point with which to compare our new qPCRs with the exact cPCRs used being a piroplasmid specific (*B. gibsoni* and *B. vogeli* inclusive) *18S* targeting nested PCR [44], a *H. canis* specific *18S* targeting PCR [45] and separate *A. platys* [46], *E. canis* [47] and *Mycoplasma* genus [48] specific *16S* targeting PCRs.

## 3. Results

### 3.1. Optimisation of Multiplex qPCRs for Canine VBPs

**Figure 1:**
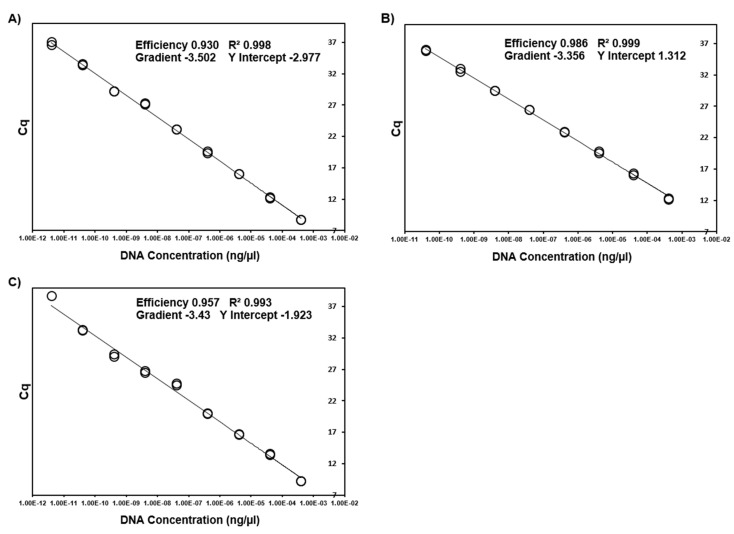
Standard curves generated from 12-fold serial dilutions of target gBlock Gene Fragments from the apicomplexans; *B. vogeli* (**A**), *B. gibsoni* (**B**) and *H. canis* (**C**).

**Figure 2:**
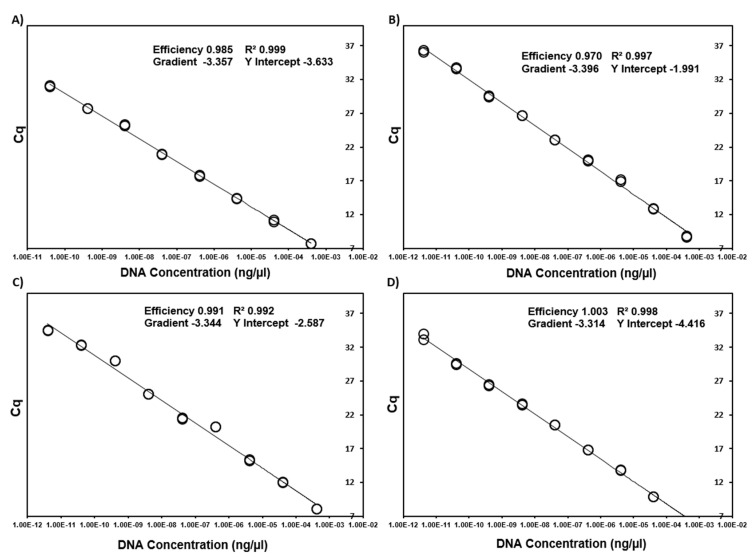
Standard curves generated from 12-fold serial dilutions of target gBlock Gene Fragments for *A. platys* (**A**), *E. canis* (**B**), *Mycoplasma* spp. (**C**) and mammalian mtDNA (**D**).

**Figure 3:**
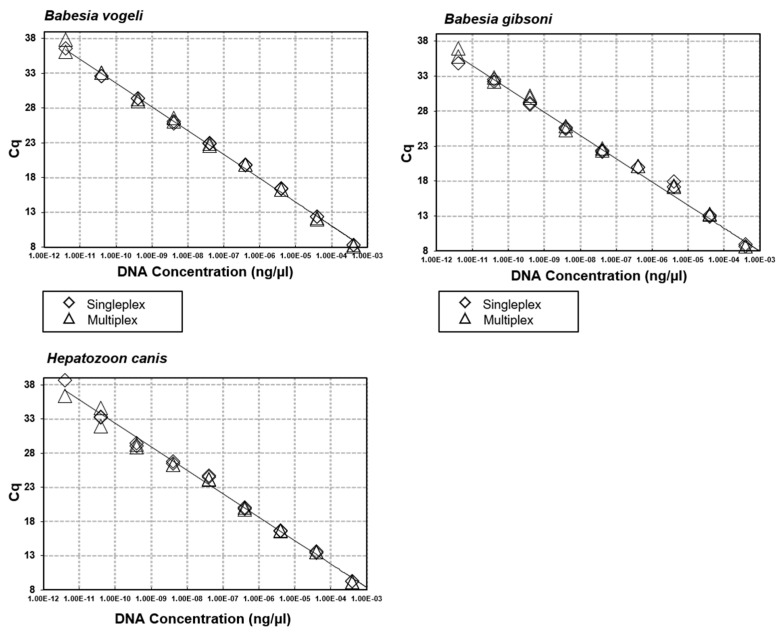
Singleplex and multiplex qPCR efficiencies. Optimisation and comparison of the sensitivity and efficiency of each singleplex and multiplex qPCR using gBlock Gene Fragment controls for *B. vogeli*, *B. gibsoni* and *H. canis*.

**Figure 4:**
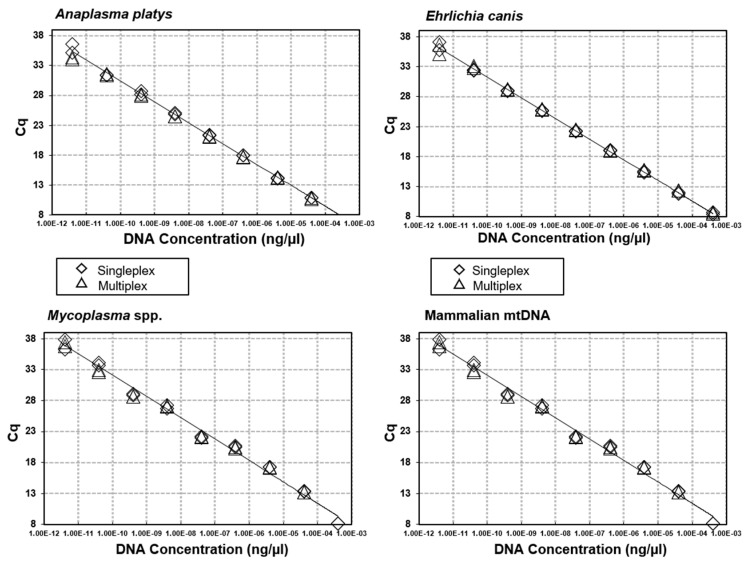
Singleplex and multiplex qPCR efficiencies. Optimisation and comparison of the sensitivity and efficiency of each singleplex and multiplex qPCR using gBlock Gene Fragment controls for *A. platys*, *E. canis*, *Mycoplasma* spp. and mammalian mtDNA.

Via iterative testing of primer and probe concentrations, optimal concentrations were identified (Table 1) that produced the highest qPCR efficiencies in both a singleplex and multiplex format. Efficiencies ranged from 93% (*B. vogeli*) to 100% (mammalian mtDNA) with an R^2^ from between 0.992 to 0.999 and slope from −3.314 to −3.502 (Figure 1 and Figure 2). When singleplex reactions were added into a quadruplex format no changes were observed in reaction efficiencies, intra-reaction reproducibility and analytical sensitivity (Figure 3 and Figure 4). No cross-reactivity was observed on gBlock synthetic DNA fragments for any of the target pathogens in either of the two quadruplex reactions.

### 3.2. Sensitivity & Specificity of Multiplex qPCRs for Canine VBPs

Analytical sensitivity of our multiplex qPCRs was assessed by identifying the lowest possible gBlock DNA concentration that was detectable, along with the corresponding Cq value for that concentration, for each pathogen targeted. Mean Cq values associated with minimal detection limits were 36.83 (S.D. ± 0.2) for *B. vogeli*, 37.32 (S.D. ± 0.3) for *B. gibsoni*, 38.68 for *H. canis*, 35.86 (S.D. ± 0.7) for *A. platys*, 36.42 (S.D. ± 0.6) for *E. canis*, 37.06 (S.D. ± 0.8) for haemotropic *Mycoplasma* spp. and 36.85 (S.D. ± 0.4) for mammalian mtDNA corresponding to 1.2 × 10^−4^ fg of target gBlock DNA for *B. gibsoni*, 1.08 × 10^−5^ fg for *B. vogeli*, 2.9 × 10^−4^ fg for *H. canis*, 2.8 × 10^−4^ fg for *A. platys*, 7.8 × 10^−4^ fg for *E. canis*, 5.2 × 10^−4^ fg for haemotropic *Mycoplasma* spp. and 5.2 × 10^−3^ fg for mammalian mtDNA.

High analytical specificity was confirmed via the absence of any cross-reactivity on off-target genomic DNA samples. Diagnostic sensitivity of our novel qPCRs, when using the merged cPCR and NGS metabarcoding results as a gold standard, was 95.5% (84/88; 95% CI 88.9–98.2), whilst diagnostic specificity was 91.7% (11/12; 95% CI 64.6–98.5).

### 3.3. Performance of Multiplex qPCRs on Field Samples

The ability of our newly developed multiplex qPCRs to accurately and sensitively detect the targeted canine VBPs from 100 field samples was assessed using a set of blood-extracted DNA from stray dogs in Thailand, previously characterised by cPCR and NGS metabarcoding [13,20]. The results of these two tests provided an accurate benchmark with which to compare the field sensitivity and specificity of our novel qPCR protocol. For all sample batches tested via qPCR, mammalian DNA, EHV and gBlock positive controls amplified within the expected Cq values, demonstrating successful DNA extraction and the absence of qPCR inhibition. Negative control samples were always absent of any amplification (with no Cq values), demonstrating that no reaction cross-contamination had occurred.

Comparisons of cPCR vs. qPCR performance for these 100 Thai field samples are shown in Table 2, whilst comparisons of NGS metabarcoding results against those acquired by our novel qPCR are displayed in Table 3. Overall our multiplex qPCR detected more VBPs in the Thai canine cohort than the cPCR assays for all pathogens apart from haemotropic *Mycoplasma* spp. with total agreement ranging from 72% for *H. canis* to 98% for haemotropic *Mycoplasma* spp. Kappa agreement for VBP detection was high for both *B. vogeli* and haemotropic *Mycoplasma* spp., substantial for *E. canis*, moderate for *A. platys* and fair for *H. canis* with lower levels of agreement observed for these latter three pathogens due to the qPCR detecting numerous VBP positive samples that the cPCR assays had missed. For example, with respect to *H. canis* the multiplex qPCR outperformed the cPCR detecting 24% more infections, whilst in contrast, agreement for *H. canis* detection was substantial between the NGS and qPCR methodologies (Table 2).

Agreement between a previously developed NGS metabarcoding assay and the multiplex qPCR assays is shown in Table 3. Methodological agreement was high for the detection of *A. platys*, haemotropic *Mycoplasma* spp. and *B. vogeli*, whilst substantial agreement was identified for *E. canis* and *H. canis* with the qPCR assay detecting a greater number of infections for these two pathogens than the NGS protocol. Agreement between the NGS and qPCR assays was higher for the detection of *A. platys*, *E. canis* and *H. canis* when compared to the agreement for cPCR vs. qPCR, whereas for *B. vogeli* and haemotropic *Mycoplasma* spp. detection agreement was similar to that achieved by cPCR vs. qPCR.

A kappa statistic diagnostic comparison for *B. gibsoni* could not be made given the absence of this species in the Thai field samples. Nonetheless, a batch of twelve samples from stray dogs in China that were confirmed for the presence of *B. gibsoni* via cPCR and Sanger sequencing (100% query cover and nucleotide identity to accession #: MN134517.1) were also tested using the relevant apicomplexan quadruplex qPCR, with Cq values ranging from 26 to 30.

## 4. Discussion

In the present study, we have demonstrated the development, optimisation and validation of two novel quadruplex qPCR assays for the simultaneous detection of six different canine VBPs. Such diagnostic tools will significantly aid researchers in the accurate diagnosis of common canine VBPs in the Asia-Pacific, permitting epidemiological surveillance of these pathogens in a manner that can easily be scaled up into a high-throughput format.

Clinical signs presented by dogs infected with common VBPs are typically shared by numerous different aetiological agents, whilst successful treatment may vary greatly depending on the species implicated [8,9,10]. Specific and sensitive diagnosis of the exact pathogen responsible is critical, with our newly developed multiplex qPCR being capable of meeting such requirements, whilst also providing relatively rapid diagnosis compared with the time and labour needed to conduct microscopy and cPCR. Additionally, our new qPCR protocol obviates many of the challenges associated with microscopy-based analysis such as low sensitivity and the high-levels of expertise required by the microscopist to accurately identify VBPs, particularly in individuals that are coinfected with species that may be morphologically similar [33].

Real-time PCR assays for the detection of *A. platys*, *E. canis*, haemotropic *Mycoplasma* spp., *B. vogeli*, *B. gibsoni* and *H. canis* have been developed previously, although none of these methods have targeted all these VBP agents simultaneously and only a proportion have utilised highly specific, Taq-Man probe-based chemistry [9,24,25,26,28,32,38,47,49,50,51,52,53,54,55].

Many of the aforementioned VBPs are also common across the Asia-Pacific, with some species demonstrating changing distributions that mean accurate surveillance is crucial to assess changing risks to local dog populations. For example, *E. canis* has been identified molecularly in 25.5% of dogs in Malaysia [56], 21.8% in Cambodia [57] and 3.9–40% in Thailand [20,58] with further serological evidence of this pathogen from canines in China, Indonesia, the Philippines, Singapore, Taiwan and Vietnam [12]. In addition, this pathogen has now been found in Australia, a country which was previously considered *E. canis* free but that is now grappling with growing outbreaks in the north, west and centre of the country [22,41,59].

*Anaplasma platys*, haemotropic *Mycoplasma* spp., *H. canis* and *Babesia* spp. have also been found throughout the Asia-Pacific with apicomplexan species such as *B. vogeli* found in dogs at a prevalence as high as 32.7% in Cambodia [57] and 9.4% in Thailand [58], whilst the more pathogenic *B. gibsoni* has been identified less commonly in countries such as China, Singapore [12] and Australia [18,60]. *Hepatozoon canis* has also been found parasitising large numbers of canines across the region [13,57,58] and again there has been recent evidence of introduction of this species into Australia where previously it was not considered endemic [61]. Such cases emphasise the critical need for effective pathogen diagnostics alongside strong biosecurity policies to prevent the introduction of novel VBPs to a previously non-endemic country and thereby mitigate the risk to naïve local dog populations that may be particularly susceptible.

Analytical sensitivity demonstrated our qPCRs as capable of detecting DNA quantities as low as 1.08 × 10^−5^ fg (*B. vogeli*), whilst singleplex reaction efficiencies were high, ranging from 93% to 100% with little difference observed when used in a multiplex format. Analytical specificity of our qPCRs was evaluated through testing on a range of common canine VBP positive blood DNA samples (previously confirmed using cPCR and Sanger sequencing). No off-target amplification was observed for any of these positive control samples, likely aided by the inclusion of LNA bases, as well as thorough in silico testing of qPCR primers and probes to ensure any chance of off-target binding was kept to a minimum [62].

Assessment of our qPCRs’ diagnostic sensitivity and specificity against a set of samples from dogs in Thailand previously characterised via NGS and cPCR methods, demonstrated a high sensitivity at 95.5% in conjunction with good diagnostic specificity at 91.7%. Diagnostic sensitivity and specificity were calculated using merged cPCR and NGS metabarcoding results as a gold standard, given the high concordance between these methods and that NGS analysis has previously been demonstrated to be highly sensitive for detection of some VBPs, particularly when compared to microscopy [13,20,63].

Our new qPCR assays detected more VBP infections than nearly all the cPCR assays used and found similar numbers to those identified by NGS metabarcoding. Kappa agreement between cPCR and qPCR was fair for *H. canis*, moderate for *A. platys* and substantial for *E. canis* with qPCR identifying more infections than cPCR for all pathogens except haemotropic *Mycoplasma* spp. Superior sensitivity of qPCR over cPCR assays, alike to the data found in our study, has been demonstrated previously, including direct demonstration of lower limits of detection for qPCR over cPCR [34,35,64]. In the present study, differences in sensitivity between cPCR and qPCR for each pathogen are likely due to the method of cPCR visualisation, e.g., gel electrophoresis used in the present study has poorer sensitivity than capillary electrophoresis as well as the particular cPCR assays used, each of which makes use of different reaction conditions and gene target regions [65]. Such differences in diagnostic sensitivity are non-trivial and may have large implications for the results of epidemiological surveys. For example, prior studies have found 25.5% of Malaysian dogs [56], 21.8% of Cambodian dogs [57] and 3.9% of Thai dogs [58] positive for *E. canis* via cPCR, whilst in this study qPCR methods found many more dogs positive, at 46%. Hence, whether such disparity represents a true difference in prevalence or is an artefact of differences in diagnostic sensitivity is impossible to discern.

Concordance between the NGS metabarcoding method and our novel qPCRs was higher overall than the comparison against cPCR. Agreement was high for the VBPs *A. platys*, *B. vogeli* and haemotropic *Mycoplasma* spp. with substantial agreement for detection of *E. canis* and *H. canis*. For the pathogens *E. canis*, *H. canis* and *B. vogeli* our qPCRs identified slightly more infections than the NGS method, although differences were negligible. Detection by qPCR of particular pathogens has been shown to be more sensitive than NGS methods in certain circumstances, whilst also being dependent on the number of samples multiplexed within a given NGS run [66,67].

There was also a small proportion of samples found positive by NGS and negative by qPCR for all pathogens targeted. As many samples are multiplexed and analysed together during NGS protocols there can be occasional Illumina index cross-talk, misreading or sequence hybridisation errors during amplification and deep-sequencing [68,69]. These facets of Illumina sequencing can lead to reads that were present in one sample appearing to be present in another, thus providing a potential source of false positives that may have generated the discordance between our NGS and qPCR methods [68].

Despite the high sensitivity and specificity demonstrated by our novel qPCR assays, there are some inherent limitations with molecular-based diagnostic testing that users should be cognizant of. Any molecular assay requires the circulation of pathogen DNA in the body compartment to be tested to be able to identify an active infection [36,70]. This may not always be the case in asymptomatic or chronic infections where the presence of circulating pathogens in the bloodstream may be cyclical or low if the host has mounted a successful immune response [70,71]. Additionally, our qPCRs’ diagnostic specificity of 91.7% indicates that it may be prone to occasional false positive results, a factor that might be alleviated by the use of three or four replicates per sample that could improve qPCR precision [72]. Therefore, taking both factors together, we would advise that in a clinical setting additional serology-based tests that detect circulating pathogen-specific antibody, may also need to be conducted to ensure an accurate diagnosis is performed.

Our novel assays detailed herein may not only be a useful diagnostic method for use in a veterinary context but also for pulling apart canine VBP transmission dynamics in other relevant lifecycle hosts, such as arthropod vectors or non-domesticated wildlife. For example, our qPCRs could be used to test samples from wild canids that are known to maintain reservoirs of some VBPs [73,74], whilst a modification to the assay’s DNA extraction control to detect invertebrate mtDNA could allow the qPCRs to surveil for canine VBP prevalence in collected tick and flea vectors as well.

In conclusion, our two newly developed quadruplex qPCR assays for the detection of six common canine VBPs found across the Asia-Pacific demonstrated higher diagnostic sensitivity when compared to cPCR methods and were able to detect coinfections that the individual cPCRs were not able to do. Furthermore, our novel qPCRs demonstrated comparable, if not superior, diagnostic sensitivity when compared to NGS metabarcoding methodologies, being able to achieve similar results at a fraction of the cost and labour. Overall, we envision that these new qPCRs will greatly assist researchers in conducting future epidemiological surveillance of canine VBPs over a geographic region stretching from China, through Southeast Asia to Australia where the target pathogens may be found. Our methods may provide tools to predict and uncover emerging VBP threats to previously non-endemic areas at a time when global climate disruption, increasing movement and globalisation, rapidly change patterns of worldwide disease prevalence [75].

## Figures and Tables

**Table 1:** Oligonucleotide primers and probes, including fluorophores, for two quadruplex assays for canine VBP detection, with a + denoting a proceeding locked nucleic acid (LNA) base. Final reagent concentrations in reactions are shown.

Target	Primers and Probes	Sequence (5′–3′)	Gene Target	Size (bp)	Final Conc. (nm)	Source
*B. gibsoni*, *B. vogeli* & *H. canis*	Api-F	AGGAAGWTT RAG GCAATAACAG	*18S rRNA*	180	700	This study
	Api-R	CTA GGCATT CCT CGT TMAWGATT	*18S rRNA*	180	700	This study
*B. gibsoni*	Bgib-Probe	/56-FAM/TATCCCTG G/ZEN/CCGAGAG GTC C/3IABkFQ/	*18S rRNA*		60	This study
*B. vogeli*	Bvog-affinity-Probe	/5HEX/AG +T+T+G +TTC +C+TYGG/3IABkFQ/	*18S rRNA*		400	This study
*H. canis*	Hcan-probe	/56-ROXN/TGAATGTG CATCGTGATGGGAAT AGA/3IAbRQSp/	*18S rRNA*		100	This study
Equine Herpes Virus 4	EHV-F	GATGACACTAGCG ACTTCGA	*gB* gene	80	80	[42]
	EHV-R	CAGGGCAGAAACC ATAGACA	*gB* gene	80	80	[42]
	EHV-Probe	/5Cy5/TTTCGCGT G/TAO/C CTC CTCCAG/3IAbRQSp/	*gB* gene		200	[42]
*A. platys* & *E. canis*	Ehr/Ana-F	TCA GAA CGA ACGCTGGC	*16S rRNA*	145–149	200	This study
	Ehr/Ana-R	CACCATTTCTAR TGGCTATYCC	*16S rRNA*	145–149	200	This study
*A. platys*	APlat-Probe	/56-FAM/CG GAT TT+T +TGTCGTAGCTTG CT+ATG/3IABkFQ/	*16S rRNA*		50	This study
*E. canis*	ECan-ALT-Probe	/56-ROXN/TA GCC TCT GGCTAT A+G+G AAA TTGT/3IAbRQSp/	*16S rRNA*		150	This study
Haemotropic *Mycoplasma* spp.	Myco-F-D1	CAM GTC AAG TCATCATGCCC	*16S rRNA*	134	250	This study
	Myco-R-Mod1	CGA ATT GCA GCC TYYTAT CC	*16S rRNA*	134	250	This study
	Myco-ALT-Probe	/5HEX/TG +CAAA+C+G TGCTACAATGG/3IABkFQ/	*16S rRNA*	134	200	This study
Mammalian mtDNA	Mam-F	CGACCTCGATGT TGGATCAG	mtDNA	92	100	[35]
	Mam-R	GAACTCAGA TCA CGT AGG ACT TT	mtDNA	92	100	[35]
	Mam-Probe	/5Cy5/CCTAATGGTGC AGCAGC+TA+TTAA GG/3IAbRQSp/	mtDNA	92	50	This study

**Table 2:** cPCR vs. multiplex qPCR agreement statistics for five VBPs. POS = positive, NEG = negative, SE = standard error. Kappa agreement level defined as poor if coefficient (*k*) is ≤ 0.20, fair agreement if 0.21 ≤ *k* ≤ 0.40, moderate agreement if 0.41 ≤ *k* ≤ 0.60, substantial agreement if 0.61 ≤ *k* ≤ 0.80 and high agreement if *k* > 0.81.

VBP	cPCR	qPCR		Total Agreement (%)	Kappa (Agreement)	Kappa SE
		NEG	POS			
*A. platys*	NEG	75	13	87	0.581 (moderate)	0.098
	POS	0	12			
*E. canis*	NEG	53	14	85	0.692 (substantial)	0.071
	POS	1	32			
*Mycoplasma* spp.	NEG	61	0	98	0.958 (high)	0.03
	POS	2	37			
*B. vogeli*	NEG	86	1	99	0.957 (high)	0.043
	POS	0	13			
*H. canis*	NEG	57	26	72	0.365 (fair)	0.084
	POS	2	15			

**Table 3:** NGS vs. multiplex qPCR agreement statistics for five VBPs. POS = positive, NEG = negative, SE = standard error. Kappa agreement level defined as poor if coefficient (*k*) is ≤ 0.20, fair agreement if 0.21 ≤ *k* ≤ 0.40, moderate agreement if 0.41 ≤ *k* ≤ 0.60, substantial agreement if 0.61 ≤ *k* ≤ 0.80 and high agreement if *k* > 0.81.

VBP	NGS	qPCR		Total Agreement (%)	Kappa (Agreement)	Kappa SE
		NEG	POS			
*A. platys*	NEG	73	2	96	0.893 (high)	0.052
	POS	2	23			
*E. canis*	NEG	51	8	89	0.777 (substantial)	0.063
	POS	3	38			
*Mycoplasma* spp.	NEG	60	2	95	0.893 (high)	0.046
	POS	3	35			
*B. vogeli*	NEG	85	2	97	0.872 (high)	0.073
	POS	1	12			
*H. canis*	NEG	55	7	89	0.770 (substantial)	0.065
	POS	4	34			

## Data Availability

The datasets supporting the conclusions of this article are included within the article (and its Supplementary files).

## Supplementary Materials

The following are available online at https://www.mdpi.com/article/10.3390/microorganisms9051092/s1, Supplementary File S1: Target and non-target bacterial and protozoan *16S rRNA* and *18S rRNA* sequence accessions used in multiplex qPCR designs. Supplementary File S2: Sequence alignments for qPCR quadruplex designs.
