# Supplementary material for: Novel High-Throughput Multiplex qPCRs for the Detection of Canine Vector-Borne Pathogens in the Asia-Pacific
Source: Microorganisms. 2021 May 19;9(5):1092. doi: 10.3390/microorganisms9051092 (PMC8161336; doi:10.3390/microorganisms9051092)
Supplement: Supplementary file 1 [file microorganisms-09-01092-s001.zip › microorganisms-1232084-S/Supplementary File 1 with Species used for qPCR designs.pdf]

Supplementary file 1. Target and non-target bacterial and protozoan 16S rRNA and 18S rRNA sequence accessions used in multiplex qPCR designs.

To ensure specificity of Taq-Man probes to the target pathogen species a range of non-target sequences representing common pathogenic species as well as reagent and environmental bacterial contaminants were included.

**Bacterial quadruplex design**		**Apicomplexan quadruplex design**	
**Target species**	**NCBI accession #**	**Target species**	**NCBI accession #**
Anaplasma platys	EF139459.1	*Babesia gibsoni*	KC461261
	LC269822.1		KF511956.1
	KU586001.1		KC954653.1
	MK814413.1	*Babesia vogeli*	AY371198.1
	LC269820.1		KY290979.1
	AF536828.1		AY371196.1
	MK121782.1		HM590440.1
Ehrlichia canis	U26740.1	*Hepatozoon canis*	KX712129.1
	AF536827.1		AY150067
	EU106856.1		DQ111754.1
	EU143637.1		KJ634654.1
Mycoplasma haemocanis	KY117659.1		
	AY150973.1		
	EF416568.1		
Candidatus Mycoplasma haematoparvum	AY532390.1		
	KY117661.1		
	GQ129114.1		
Mycoplasma haemofelis	EU145745.1		
Candidatus Mycoplasma haemominutum	AY150981.1		
Candidatus Mycoplasma turicensis	DQ464423.1		
Mycoplasma coccoides	AY171918.1		
Mycoplasma suis	EU603330.1		
**Non-target species**	**NCBI accession #**	**Non-target species**	**NCBI accession #**
Acinetobacter viviani	NR148847.1	*Babesia canis*	L19079.1
Anaerobacillus alkalilacustris	NR115854.1	*Babesia conradae*	AF158702.1
Anaplasma phagocytophilum	DQ458808.2	*Babesia divergens*	LC477143.1
Bartonella clarridgeiae	NR036961.1	*Babesia microti*	AB241631.1
Bartonella henselae	L35101.1	*Babesia ovis*	AY998123.1
Bartonella vinsonii	DQ228135.1	*Hammondia hammondi*	KT184369.1
Borrelia burgdorferi	GQ478290.1	*Hammondia heydorni*	KT184370.1

Bradyrhizobium centrosematis	KC247115.1	*Hepatozoon americanum*	AF176836
Candidatus Neoehrlichia mikurensis	AB213021.1	*Hepatozoon felis*	KM435071
Candidatus Rickettsia asemboensis	JN315967.1	*Hepatozoon sipedon*	JN181157
Corynebacterium aurimucosum	AY536426.1	*Leishmania donovani*	XR_002966730.1
Ehrlichia chaffeensis	AF147752.2	*Leishmania infantum*	AJ634343.1
Ehrlichia ewingii	NR044747.1	*Plasmodium berghei*	AJ243513
Enhydrobacter aerosaccus	AB641400.1	*Plasmodium ovale*	KF018656
Pseudomonas syringae	AB001448.1	*Sarcocystis neurona*	KT184371.1
Rickettsia conorii	NR041934.1	*Theileria annulata*	EU083801
Rickettsia felis	DQ102712.1	*Theileria buffeli*	DQ104611
Rickettsia rickettsii	L36217.1	*Theileria ovis*	AY260172
Rickettsia typhi	NR118679.1	*Theileria sergenti*	EU083802
Sediminibacterium aquarii	NR152667.1	*Theileria sinensis*	KF559355
Sphingomonas ginsengisoli	AB245347.1	*Toxoplasma gondii*	L24381.1
Staphylococcus aureus	EF463060.1	*Trypanosoma evansi*	AJ009154.1
Stenotrophomonas maltophilia	FJ657669.1		
Streptococcus lutetiensis	NR037096.1		
