# Supplementary material for: Novel High-Throughput Multiplex qPCRs for the Detection of Canine Vector-Borne Pathogens in the Asia-Pacific
Source: Microorganisms. 2021 May 19;9(5):1092. doi: 10.3390/microorganisms9051092 (PMC8161336; doi:10.3390/microorganisms9051092)

Supplementary file 2. Sequence alignments for qPCR quadruplex designs.

Alignment of *B. vogeli, B. gibsoni* and *H. canis* 18S rRNA sequences with relevant primer pair (green) and probes (red, blue, yellow). All alignments made in Geneious Prime v. 2021.0.3 (Biomatters Ltd.).

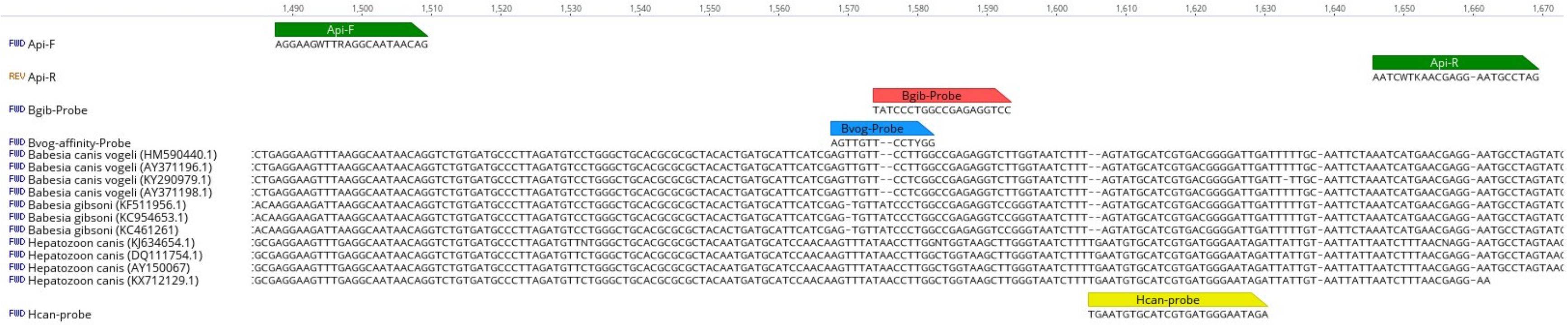

Alignment of *A. platys* and *E. canis* 16S rRNA sequences with relevant primer pair (green) and probes (pink, orange).

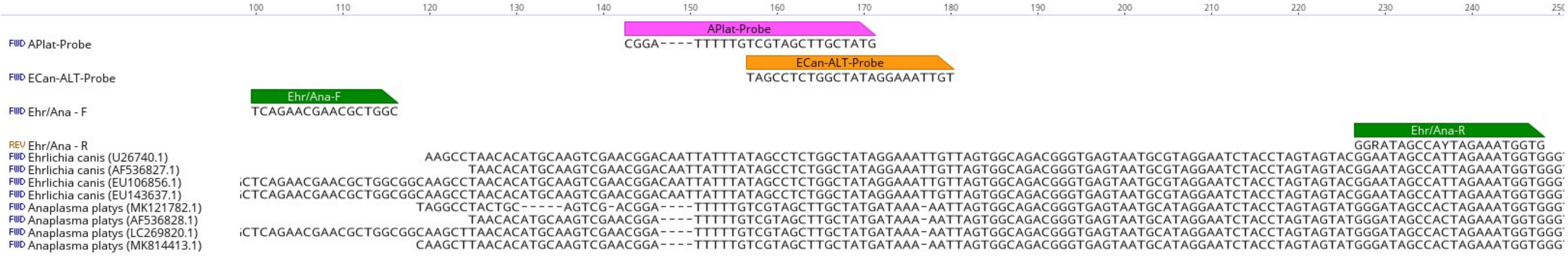

Alignment of canine infecting and non-canine infecting haemotropic *Mycoplasma* species' 16S rRNA sequences with relevant primer pair (green) and probe (purple).

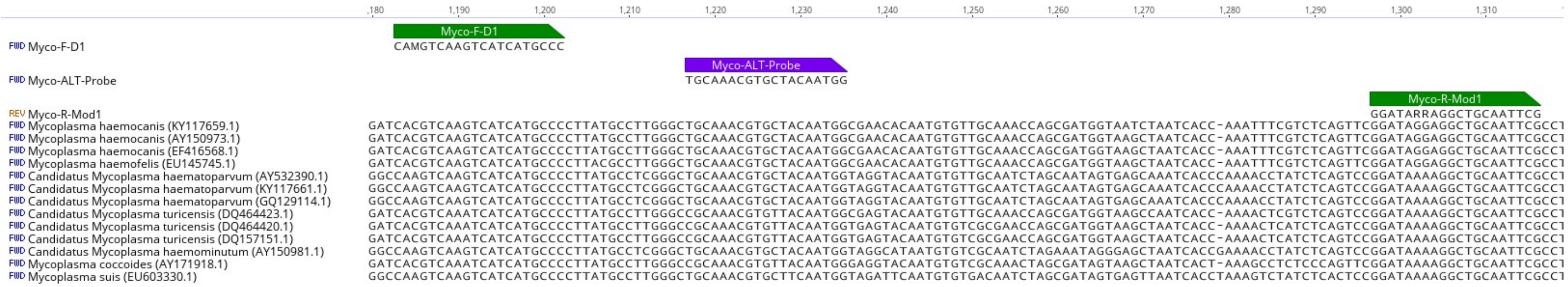

Supplement: Supplementary file 1 [file microorganisms-09-01092-s001.zip › microorganisms-1232084-S/Supplementary File 2 with qPCR design alignments.pdf]
